# Vascular microarray profiling in two models of hypertension identifies caveolin-1, Rgs2 and Rgs5 as antihypertensive targets

**DOI:** 10.1186/1471-2164-8-404

**Published:** 2007-11-07

**Authors:** T Hilton Grayson, Stephen J Ohms, Therese D Brackenbury, Kate R Meaney, Kaiman Peng, Yvonne E Pittelkow, Susan R Wilson, Shaun L Sandow, Caryl E Hill

**Affiliations:** 1 Division of Neuroscience, John Curtin School of Medical Research, Australian National University, Canberra, Australia; 2 Australian Cancer Research Foundation Biomolecular Resource Facility, John Curtin School of Medical Research, Australian National University, Canberra, Australia; 3 Centre for Bioinformation Science, Mathematical Sciences Institute, Australian National University, Canberra, Australia; 4 School of Medical Sciences, University of New South Wales, Sydney, Australia

## Abstract

**Background:**

Hypertension is a complex disease with many contributory genetic and environmental factors. We aimed to identify common targets for therapy by gene expression profiling of a resistance artery taken from animals representing two different models of hypertension. We studied gene expression and morphology of a saphenous artery branch in normotensive WKY rats, spontaneously hypertensive rats (SHR) and adrenocorticotropic hormone (ACTH)-induced hypertensive rats.

**Results:**

Differential remodeling of arteries occurred in SHR and ACTH-treated rats, involving changes in both smooth muscle and endothelium. Increased expression of smooth muscle cell growth promoters and decreased expression of growth suppressors confirmed smooth muscle cell proliferation in SHR but not in ACTH. Differential gene expression between arteries from the two hypertensive models extended to the renin-angiotensin system, MAP kinase pathways, mitochondrial activity, lipid metabolism, extracellular matrix and calcium handling. In contrast, arteries from both hypertensive models exhibited significant increases in caveolin-1 expression and decreases in the regulators of G-protein signalling, Rgs2 and Rgs5. Increased protein expression of caveolin-1 and increased incidence of caveolae was found in both smooth muscle and endothelial cells of arteries from both hypertensive models.

**Conclusion:**

We conclude that the majority of differences in gene expression found in the saphenous artery taken from rats with two different forms of hypertension reflect distinctive morphological and physiological alterations. However, changes in common to caveolin-1 expression and G protein signalling, through attenuation of Rgs2 and Rgs5, may contribute to hypertension through augmentation of vasoconstrictor pathways and provide potential targets for common drug development.

## Background

Hypertension is a complex condition with many contributory genetic and environmental factors [1], which have constrained identification of the genes, biochemical pathways and pathophysiological processes that accompany its development. Hypertension is a critical risk factor for stroke, renal failure and cardiovascular disease and has been associated with a range of well recognized complications, including congestive heart failure, left ventricular hypertrophy, and atherosclerosis, and with more subtle structural and functional changes to target organs and tissues occurring as a consequence of enhanced salt sensitivity [2], enhanced angiotensin II sensitivity [3] and structural modifications to the arterial wall [4,5]. Genetic factors are thought to partly account for structural changes by directly influencing the quantity, density and organization of the extracellular matrix and also indirectly through association with risk factors such as age, gender, blood pressure, total and high-density lipoprotein cholesterol, smoking and diabetes [6].

A variety of both genetic, such as the spontaneously hypertensive rat (SHR), and nongenetic, such as glucocorticoid-induced hypertension, animal models of hypertension have been developed [7]. Administration of adrenocorticotropic hormone (ACTH) to rats provides a quick and reproducible means of inducing hypertension by stimulating the adrenal production of corticosterone [8]. ACTH-induced hypertension was originally thought to involve altered kidney function and loss of control of blood volume [9] but is now known to involve disruption of the nitric oxide synthase (NOS) pathway at various points, affecting NO bioavailability, oxidative stress and function of the endothelium [10,11]. The genetically hypertensive SHR is a renin-angiotensin system-dependent model of hypertension that also exhibits oxidative stress and disrupted endothelial NOS (eNOS) activity [12,13]. Such convergence in effectors holds the promise of some overlap in causative factors.

Hypertension is generally characterized by remodeling of arteries, such as thickening of the media and intima, reduced lumen diameter and reduced distensibility [14]. Endothelial dysfunction is also a characteristic of hypertension and alterations to endothelial morphology and density have been described [15-17]. Resistance vessels have a primary role in autoregulation of the blood supply to the vascular bed and changes to their structure and function are crucial to the onset of target organ damage [4]. Angiotensin II has a prominent role in affecting resistance vessels as a pro-inflammatory stimulator of signaling pathways promoting vascular growth, remodeling and oxidative stress [18,19]. Both hypertrophic and eutrophic remodeling have been documented in arteries of SHR, perhaps related to the size of the vessel and intraluminal pressures experienced [20-23]. Whether similar changes to resistance vessels accompany ACTH-induced hypertension is not known. Microarray expression profiling offers a means of studying the modifications to global gene expression that accompany these events [24] and hence may provide novel insights into potential targets for common therapeutic intervention. The aim of the present study was therefore to compare gene expression and morphology of a resistance artery from adult normotensive Wistar-Kyoto (WKY) rats, SHR and ACTH-induced hypertensive rats; the latter two as representatives of renin-angiotensin-dependent and steroid-dependent hypertension, respectively. Although the majority of genetic changes differed between the two hypertensive models, changes in common were found to caveolin-1 expression and the regulators of G protein signalling, Rgs2 and Rgs5.

## Results

Blood pressure of SHR and ACTH-treated rats was significantly elevated over that of age-matched WKY rats (Table 1).

**Table 1:** Morphological characteristics of primary branches of the saphenous artery

Parameter	WKY	ACTH	SHR
Systolic BP (mmHg)	123 ± 5 (5)	146 ± 3* (10)	187 ± 4* (9)
Circumference (μm)	745 ± 36 (4)	628 ± 51 (7)	610 ± 22 (6)
Mean SMC layers	7.2 ± 0.1 (4)	6.7 ± 0.4 (7)	8.7 ± 0.4* (5)
Area of EC (μm^2^)	392 ± 9 (5)	438 ± 13* (6)	279 ± 4* (3)
MEGJs/EC	0.1 ± 0.08 (4)	0.06 ± 0.04 (4)	0.03 ± 0.03 (3)

Mean ± SEM; number of animals in parentheses. *p < 0.05 compared to WKY. Myoendothelial gap junctions (MEGJs) are expressed per endothelial cell (EC). Measurement of EC area from 150 cells (WKY), 76 cells (ACTH) and 45 cells (SHR). SMC = smooth muscle cell. Data for WKY from reference [49].

### Anatomy of saphenous arteries from normotensive and hypertensive rats

**Figure 1:**
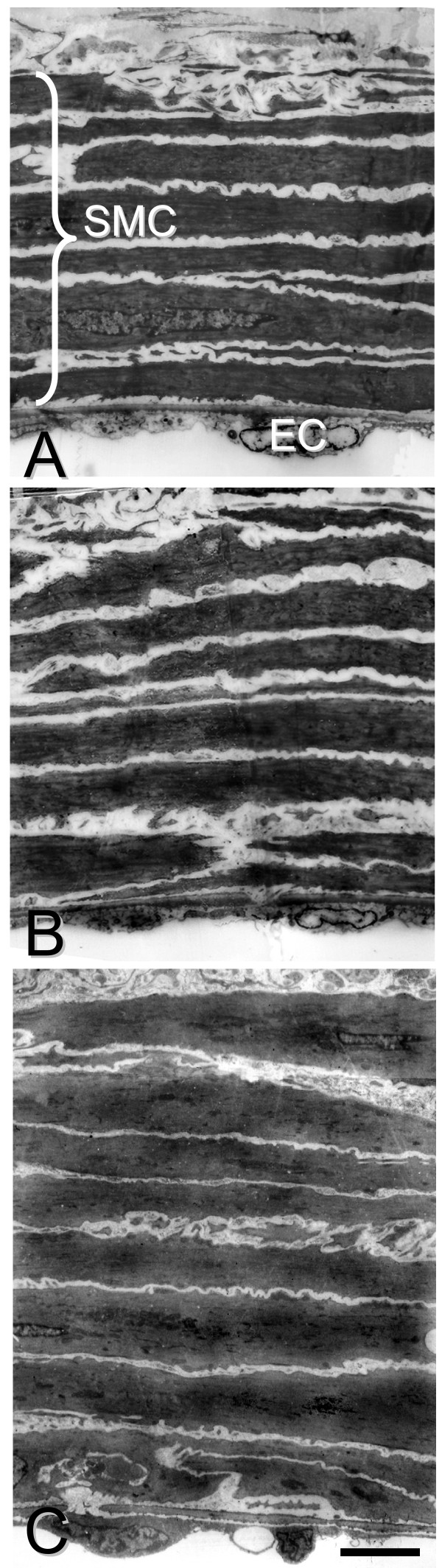
**Typical morphology of saphenous arteries from 16 week normotensive and hypertensive animals**. Fewer smooth muscle layers were present in vessels from normotensive WKY (A) and hypertensive ACTH (B) than in SHR (C). Bar, 10 μm.

The internal vessel circumference of arteries taken from both ACTH-treated rats and SHR showed a trend towards a decrease, compared with WKY, although only saphenous arteries from SHR exhibited hypertrophy of the smooth muscle cell layers (Figure 1, Table 1). In the endothelium, there was a significant reduction in the area of endothelial cells in SHR, while area was significantly increased during ACTH-treatment (Table 1). There was no significant difference between experimental groups in the density of myoendothelial gap junctions per endothelial cell, which were rarely encountered in either WKY, SHR or ACTH-treated rats (Table 1).

### Assessment of RNA degradation

The extent of RNA degradation was assessed by comparing the MAS5-derived 3'/5' and 3'/M' (middle) ratios of the housekeeping genes, GAPDH and hexokinase sample (Table 2). The 3'/5' ratios for GAPDH for all 12 samples were in the range 3–7, which is acceptable for doubly-amplified RNA, while the 3'/M' ratios for GAPDH were less than 2. Similar data were found for the 3'/5' and 3'/M ratios for hexokinase although some of the ratios tended to be greater than those for GAPDH, perhaps due to the longer hexokinase transcripts (3653 bp, versus 1268 bp for GAPDH).

**Table 2:** MAS5-derived 3'/5' and 3'/M' (in brackets) ratios of housekeeping genes.

**Array name**	**RAT-GAPDH**	**RAT-HEXOKINASE**
SHR1	3.57 (1.38)	2.55 (3.05)
SHR2	3.79 (1.32)	1.63 (1.80)
SHR3	5.16 (1.55)	3.39 (3.42)
WKY1	5.35 (1.89)	2.01 (4.23)
WKY2	5.99 (1.76)	1.61 (2.15)
WKY3b	4.91 (1.66)	8.79 (0.69)
ACTH1	3.96 (1.64)	3.56 (3.02)
ACTH2	4.52 (1.72)	4.48 (0.72)
ACTH3	3.67 (1.64)	12.54 (1.08)

### Statistical analysis of microarrays

Of the 15,878 probesets on each array, 9098 were present in all replicates of at least one experimental group (Affymetrix MAS5 Statistical Algorithm) and considered for further analysis. One-way ANOVA on each of the 9098 probesets showed significant differences in expression of 870 genes amongst the 3 experimental groups (F-test p value cutoff of 0.02) or 136 genes (p value cutoff of 0.001). The data discussed in this publication have been deposited in NCBIs Gene Expression Omnibus (GEO) at the following website [25] and are accessible through GEO Series accession number GSE8051.

**Figure 2:**
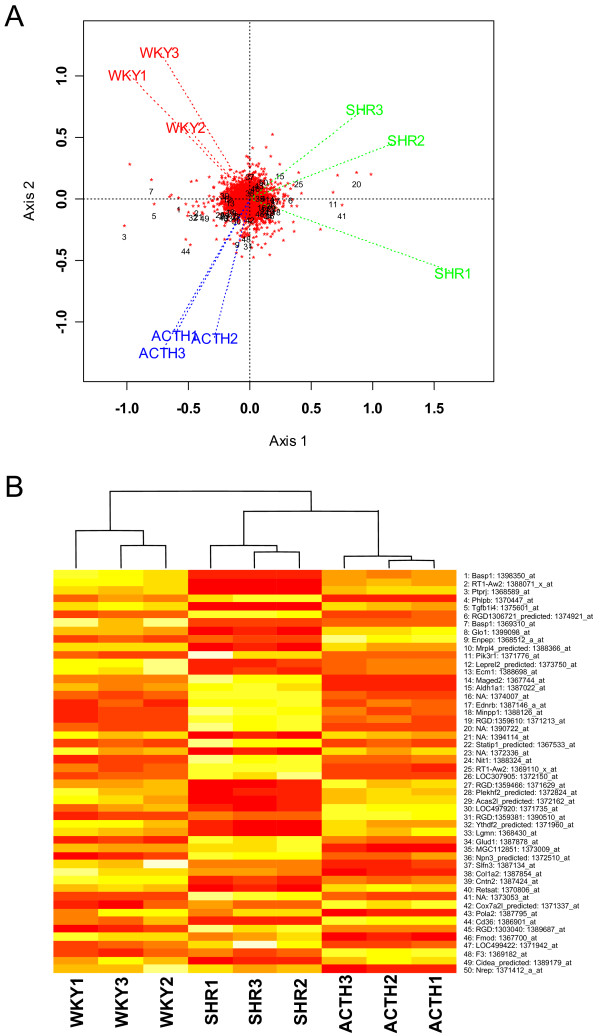
**Clustering of arrays visualised by two different methods**. A. GE-biplot of the 9098 probesets (red asterisks) with the first 50 (1 = most significant), as ranked by ANOVA, indicated. The position of each array is shown as a label joined by a dotted line to the origin. B. A heat map (white: up-regulated; red: down-regulated) using hierarchical clustering with complete linkage for the columns only. The rank order of the p-values, gene names and Affymetrix IDs are included on the right and correspond to the numbering in A.

The 50 most significant probesets found by ANOVA were superimposed on a GE-biplot of the 9098 probesets present in all arrays of at least one experimental group (Figure 2A). The locations of the probesets are shown as red asterisks with the 50 most significant genes indicated. The locations of the arrays are connected to the centre of the plot and labelled by their respective names. Figure 2A shows that the replicates within each experimental group are clustered together relative to the other groups, indicating consistency in measurement. In this genome-wide view the three experimental groups, WKY, ACTH and SHR groups are well separated, providing evidence of overall transcriptional differences between the groups.

The genes separating from the dense group in the centre of Figure 2A have relatively high variance. The labelled genes have a statistically significant difference in their means in the three groups. Generally the statistically significant genes on the left of the plot have relatively low expressions in SHR but high and approximately equal expression in WKY and ACTH. Those statistically significant genes on the right of the centre have relatively high expression values in SHR and low and approximately equal expression in WKY and ACTH. These patterns in gene expression are the dominant genome-wide patterns. However there are a number of genes which are relatively highly expressed in ACTH (eg. 9, 31, 48) compared to WKY and SHR.

The heat map of the 50 most significant genes in Figure 2B shows that the arrays cluster according to experimental groups but the experimental groups differ substantially, in agreement with clustering in the GE-biplot.

### Validation of microarray results by quantitative PCR

Eleven out of 12 genes (6 in SHR, 6 in ACTH) which showed significant variation from WKY in the microarrays were validated by quantitative PCR (Table 3). The one variant gene (adenylate cyclase3) was significantly different in the SHR by microarray, but found not to differ from WKY by quantitative PCR. In contrast, four of 14 genes (7 in SHR, 7 in ACTH) which did not show any significant difference in expression in the microarrays relative to WKY, were found to be significantly different to WKY when assayed using quantitative PCR (Table 3). Three of these genes were found to be decreased in expression relative to WKY and one gene increased in expression (Table 3). These data concord with a recent analysis of microarray data which showed a high false-negative rate, or low sensitivity, especially in the case of genes showing small fold-changes [26].

**Table 3:** Validation of microarray data using quantitative PCR

Affymetrix ID	Accession No.	Gene name	Fold-change of signal Microarray		Fold-change of signal qPCR		Validate	
			
			SHR	ACTH	SHR	ACTH	SHR	ACTH
1387128_at	NM_130779.1	Adcy3, adenylate cyclase 3	0.70*	0.67*	1.01	0.37*	No	Yes
1370131_at	NM_031556.1	Cav1, caveolin 1	2.88*	2.51*	10.76*	2.53*	Yes	Yes
1367600_at	NM_022531.1 BC061872	Des, desmin	0.38*	0.87	0.58*	0.76	Yes	Yes
1371166_at	NM_021838.2 AJ011116.1	NOS3, eNOS, endothelial nitric oxide synthase	1.26	1.00	0.89	0.72	Yes	Yes
1373499_at	BF287008 U77829	Gas5, growth arrest specific 5	0.77*	1.59*	0.18*	1.55*	Yes	Yes
1386881_at	NM_012588.1	Igfbp3, insulin-like growth factor binding protein 3	1.22	3.38*	0.87	2.07*	Yes	Yes
1375857_at	AW917760 XM_220031	Myof, myoferlin	1.64*	0.90	2.52*	1.01	Yes	Yes
1371776_at	AA819268 NM_013005	Pik3r1, PI3 kinase regulatory subunit polypeptide 1	13.21*	1.12	2.30*	2.04*	Yes	No
1368144_at 1387074_at	AF321837.1 AY043246.1	Rgs2, regulator of G-protein signaling 2	1.12 0.84	0.46* 0.52*	0.63*	0.19*	No	Yes
1370228_at	X77158	Tf, transferrin	1.44	3.40*	0.65*	1.80*	No	Yes
1370023_at	NM_021654.1 M76532	Gja4, connexin37, Cx37	0.96	0.82	0.79	1.06	Yes	Yes
1368473_at	NM_019280.1 BC070935 M83092	Gja5, connexin40, Cx40	1.09	0.95	0.73	1.36	Yes	Yes
1372002_at	AI411352 NM_012567	Gja1, connexin43, Cx43	1.27	0.96	0.36*	1.07	No	Yes

* significantly different from WKY.

Two microarray values for Rgs2 represent 2 different Affymetrix probesets.

### Differentially and co-ordinately expressed genes between the two models of hypertension

**Figure 3:**
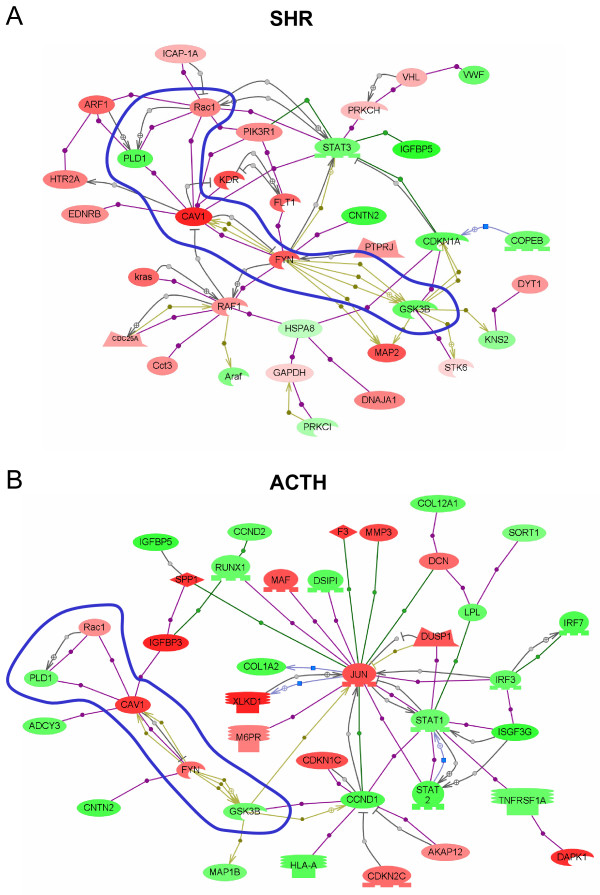
**Biological association networks of interactions between selected differentially expressed genes in SHR (A) and ACTH-induced models of hypertension (B)**. Probesets selected for input had an overall ANOVA p-value ≤ 0.02 and a t-test value ≤ 0.015 for the contrasts between SHR and WKY (483 probesets) or ACTH and WKY (377 probesets), respectively. Genes are color-coded according to the log_2_-fold change of the mean value of gene expression in SHR or ACTH compared to WKY. Red indicates up-regulated expression compared to WKY, green down-regulation. Blue shape surrounds coordinate changes in the two models. Oval = protein; oval with cutout = kinase; oval with flat base = transcription factor; polygon with flat base = phosphatase; rectangle with small base = receptor; diamond = ligand; blue square = expression; grey circle = direct regulation; purple circle = binding; green circle = promoter binding; khaki hexagon = protein modification.

Genes that were identified as significantly altered in expression by ANOVA in eight multiple comparisons of SHR and ACTH relative to WKY are given in Additional Files 1, 2, 3, 4, 5, 6, 7, 8: Tables S1–8. Genes identified in six dual comparisons of either SHR or ACTH with WKY are given in Additional Files 9, 10, 11, 12, 13, 14: Tables S9–14. Gene names, gene descriptions, biological function, Affymetrix IDs, accession numbers, expression levels and p-values are provided. The biological association networks of selected genes for each model of hypertension are shown in Figure 3A,B.

Table 4 summarises the changes in genes which relate to a range of processes or pathways which could impact on blood pressure regulation. The majority of changes reflect the specific morphological and physiological alterations occurring in the two different types of hypertension. Saphenous arteries from SHR showed upregulation of genes associated with renin-angiotensin signalling through MAP kinase ERK1/2 pathway and were characterised by upregulation of growth promoters and down regulation of growth suppressors (Table 4). In contrast, arteries from ACTH-treated animals showed upregulation of the p38MAP kinase pathway, diverse effects on growth promoters and suppressors (Table 4) and prominent involvement of the transcription factor Jun (Figure 3B). Differences were also found in expression of genes involved in mitochondrial activity, lipid metabolism, calcium handling, adenylate cyclase, ion transport and protein kinase C. In contrast, coordinate upregulation in both forms of hypertension was found for caveolin-1 and its interaction and modification by the membrane-associated tyrosine kinase Fyn (Figure 3, blue shape), and coordinate downregulation of Rgs2 and Rgs5, the regulators of G protein signalling (Tables 3, 4).

**Table 4:** Comparison of changes in gene expression in functional pathways

**Process or Pathway**	**SHR**	**ACTH**
Renin-angiotensin system	↑Ace1, ↑Atip1	
MAP kinase ERK1/2	↑Phb, ↑Raf1	↓Phb
MAP kinase p38	↓Dusp1, ↓Map4k2, ↓Map2k3	↑Dusp1
G-protein signalling	↑Gnb2, ↑Rac1 ↓Rgs2, ↓Rgs5	↑Gnb2, ↑Rac1 ↓Rgs2, ↓Rgs5
cAMP	↓Adcy3, ↓Akap12	↑Akap12 ↓Adcy2, ↓Adcy3
PKC	↑Pkcη ↓Pkcι	
Nitric oxide	↑Caveolin-1	↑Caveolin-1
Endothelins	↑EdnrB	
Reactive oxygen stress	↑Rac1 ↓Rtp801, ↓Egln3	↑Rac1 ↓Rtp801
Calcium handling	↑Anxa3, ↑Anxa4, ↑Anxa7, ↑Casq2, ↑Efhc1, ↑S100A16 ↓Anxa8, ↓Anxa11, ↓Nucb2	↑Anxa4, ↑Anxa7, ↑Efhc1
Ion transport	↑Atp1b1, ↑Kcnmb1 ↓Clcn3e, ↓Sat, ↓Ttyh1	↓Cacnb2, ↓Clcn3e, ↓Ttyh1
Mitochondrial activity	↑Aco2, ↑Mrpl3, ↑Mrpl48, ↑Mrps18b, ↑Ndufs2 ↓Frda, ↓Mrpl4, ↓Pdk2, ↓Ssbp1, ↓Timm22	↓Ndufs3, ↓Pdk2
Growth promoting	↑Minpp1, ↑P-Rex1, ↑Myof, ↑Ceacam1, ↑Ctgf, ↑Vegfr, ↑Vegfr1 ↓Aebp1, ↓Igfbp2, ↓Klf9, ↓Bmp4, ↓Igfbp5	↑Bmp4, ↑Ceacam1, ↑Ctgf, ↑Vegfr ↓Aebp1, ↓Igfbp2, ↓Igfbp5, ↓Klf9, ↓p311, ↓Pdgrfl
Growth suppressing	↓Basp1, ↓Bmp6, ↓Cst3, ↓Gas5, ↓Kank, ↓Stk11ip, ↓SerpinB5, ↓Smurf2, ↓Smoc1, ↓Tem7, ↓Tnmd	↑Gas5, ↑Igfbp3, ↑Wisp-2 ↓Basp1, ↓Tem7, ↓Tnmd
Insulin & glucose transport	↑Sord, ↑Glud1, ↑Enpp1, ↑Gsk3β ↓Bpgm, ↓Pcsk1, ↓Sort1	↑Sord, ↑Glud1 ↓Bpgm, ↓Pcsk1, ↓Sort1, ↓Gsk3β
Lipid metabolism	↑Phb ↓Decr1, ↓Acsl3, ↓Mafb	↑Acsl1, ↑Hmgcs2 ↓Lpl, ↓Phb
Extracellular matrix	↑Icap1, ↑Fyn ↓Col1A2, ↓Icam2, ↓Lox, ↓Loxl, ↓Sdc1, ↓Slit3	↑Fyn ↓Col1A1, ↓Col1A2, ↓Col3A1, ↓Col4A1, ↓Col4A2, ↓Col5A1, ↓Col8A1, ↓Col12A1, ↓Col15A1, ↓Col18A1, ↓Lox, ↓Loxl, ↓Sdc1, ↓SerpinH1, ↓Slit3
Smooth muscle-related genes	↑Acta1, ↑CaMkIId, ↑Ca3, ↑Fgl2, ↑Ldha, ↑Myof1, ↑Raf1, ↑Pcmt1 ↓Aebp1, ↓Cst3, ↓Des, ↓Gas5	↑Acta1, ↑Ca3, ↑Cnn3, ↑Gas5, ↑Igfbp3 ↓Aebp1, ↓CaMkIId, ↓Gadd45, ↓p311
Endothelium-related genes	↑Cxcl12, ↑Ecmn, ↑Gp38, ↑Hmox2, ↑Mmn1,↑Thbd ↓Recs1, ↓Tem7, ↓vWF	↑Gp38, ↑Mmn1, ↑Thbd ↓Angpt2, ↓Tem7

↑ = expression increased compared with WKY.

↓ = expression decreased compared with WKY.

See Additional Files: Tables S1–S14 for details.

### Distribution of caveolin-1 and caveolae in saphenous arteries of normotensive and hypertensive rats

**Figure 4:**
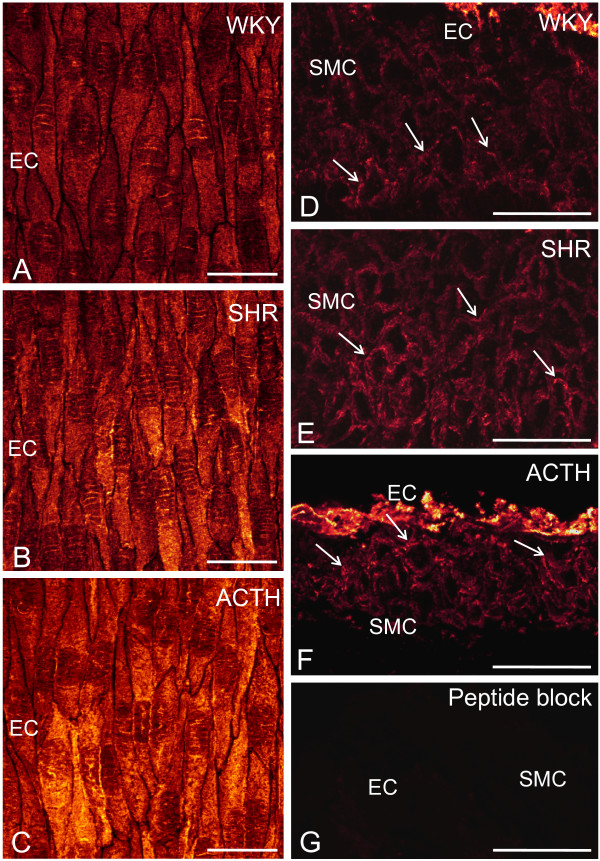
**Expression of caveolin-1 in saphenous arteries**. Whole mount preparations (A, B, C) and longitudinal sections (D, E, F) through the vessel wall show increased expression of caveolin-1 in both endothelial cells (EC: A-C) and in smooth muscle cells (SMC: D-F) of saphenous arteries taken from SHR (B, E) and ACTH (C, F), compared to WKY (A, D). Arrows indicate staining delineating the membranes of smooth muscle cells (D-F). Preincubation of the antibody with the immunogen eliminated staining in both endothelium and smooth muscle (G). Calibration bars represent 25 μm.

Expression of caveolin-1 was found in the membranes of both the endothelial and smooth muscle cells of saphenous arteries of WKY rats (Figure 4A, D, arrows). Expression varied both within and between cells in the endothelium (Figure 4A) and also throughout the muscle layers (Figure 4D). An increase in caveolin-1 expression was found in both endothelial cells and smooth muscle cells of arteries taken from both SHR and ACTH, compared to WKY rats (Figure 4B, C, compare 4A; Figure 4E, F compare 4D). Paired confocal images of either whole mounts or sections were taken with the same voltage and pinhole settings so that comparisons could be made of the extent of staining. Preincubation of the antibody with the immunogen abolished staining in endothelial and smooth muscle cells (Figure 4G).

**Figure 5:**
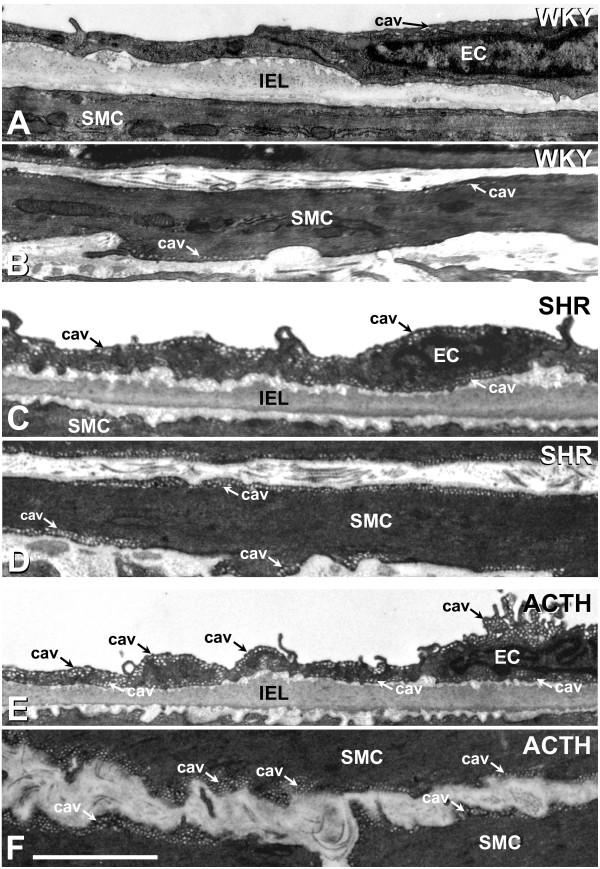
**Caveolae in endothelium and smooth muscle of saphenous arteries**. Caveolae (cav; arrows) were found along the membranes of both endothelial (EC) and smooth muscle cells (SMC) of saphenous arteries of WKY, (A, B), SHR (C, D) and ACTH-treated rats (E, F). In the SHR and ACTH-treated rats, the incidence of caveolae was increased in both cell types (C, E compare A; D, F compare B). IEL, internal elastic lamina. Bar, 5 μm.

At the ultrastructural level, caveolae were found along the membranes of both endothelial and smooth muscle cells of arteries from WKY rats (Figure 5A, B), although there were fewer on the abluminal than luminal endothelial surface. While the incidence of caveolae was increased in both cell types in the SHR (Figure 5C, D), the differential distribution in the endothelium was still apparent. In arteries from ACTH rats, a differential distribution in the endothelium was less obvious as caveolae were found in abundance on both sides of the endothelial cells (Figure 5E). Caveolae were also more abundant throughout the muscle layers of ACTH-treated rats, than of WKY rats (Figure 5F, compare 5B).

## Discussion

Comparative gene expression profiling of a resistance artery taken from rats with either renin-angiotensin-dependent or glucocorticoid-induced hypertension has identified over 800 genes showing significant changes. Statistical analysis of the microarrays showed consistency in gene expression amongst the replicates within each experimental group but evidence of substantial transcriptional differences between the two hypertensive models and the normotensive controls. The majority of these differences reflected differential vascular remodeling, as confirmed by our anatomical studies, as well as activation of specific intracellular pathways. Nevertheless, arteries from both models exhibited significantly increased expression of caveolin-1, which binds a number of important signalling molecules including endothelial nitric oxide synthase [27], and significantly decreased expression of Rgs2 and Rgs5, which terminate the signalling of the G proteins Gq and Gi/o [28]. Together these common effects would be expected to elevate blood pressure through augmentation of vasoconstrictor mechanisms.

Saphenous arteries from SHRs were characterised by hypertrophic remodeling, in contrast to the arteries from the ACTH rats in which no alterations were found in the number of smooth muscle cell layers. Similar changes have been described in other arteries of SHR [20,22,23], typical of remodeling of similarly sized resistance vessels during angiotensin II-induced hypertension [19]. The absence of hypertrophy in the saphenous artery of ACTH-treated rats suggests that inward eutrophic remodeling [14] may be more common to this form of hypertension, perhaps related to the lower blood pressures experienced in comparison with those of the SHR. In the endothelium of SHR arteries, there was a significant reduction in the area of endothelial cells, consistent with changes observed in other muscular arteries of SHR [29-31]. Surprisingly, the area of endothelial cells was increased during ACTH-treatment resulting in a decrease in overall endothelial cell numbers. Changes in gene expression were consistent with cell proliferation in angiotensin II-dependent hypertension but not in glucocorticoid-induced hypertension. Thus, expression of growth promoters was upregulated and growth inhibitors suppressed in SHR arteries, while effects on both growth promoters and inhibitors were more diverse in the ACTH arteries. A lack of change in myoendothelial gap junctions suggests that these structures are not involved in the remodeling processes in saphenous arteries in either form of hypertension.

A large number of other genetic differences in the arteries from the two hypertensive models emerged from the study. These related to lipid metabolism, mitochondrial activity, calcium handling, ion transport, the adenylate cyclase pathway and protein kinase C. Not surprisingly, saphenous arteries from SHR showed upregulation of genes consistent with renin-angiotensin hypertension, and angiotensin II signaling pathways through Raf and MEK1/2 [19]. Upregulation of growth promoters and down regulation of growth suppressors as found in the SHR samples would be expected to stimulate cell growth and proliferation, mobilise intracellular Ca^2+ ^and reactive oxygen species [18,32]. In contrast, the saphenous arteries from ACTH-treated animals showed upregulation of the p38MAP kinase pathway which is involved in inflammation and apoptosis, as well as growth and differentiation [33], accentuating mechanistic differences between the two models of hypertension. While alterations to the extracellular matrix were found in both hypertensive models, substantial changes involving multiple collagen genes were found predominantly in the ACTH model. Such changes have been linked with hypertension [34] and could impact on cytoskeletal signalling through integrin receptors.

Downstream mechanisms underlying smooth muscle contraction and calcium sensitization also varied between the two hypertensive models. Calcium/calmodulin-dependent protein kinase II (CaMkII) has an important role in regulating smooth muscle contractility by inhibiting myosin light chain kinase (MLCK) and the phosphorylation of myosin light chain [35]. In SHR, upregulation of CaMkII may shift smooth muscle contraction toward Rho-dependent mechanisms and decreased phosphatase activity. In contrast, in ACTH, CaMkII was downregulated suggesting that smooth muscle contraction could be more influenced by MLCK, in addition to Rho-dependent mechanisms.

In the face of these substantial differences in gene expression between the two hypertensive models, we found coordinate upregulation in gene expression of caveolin-1 and increased caveolin-1 protein in both endothelial and smooth muscle cells. Our ultrastructural observations of increased incidence of caveolae in these sites provides strong confirmation of the immunohistochemical findings since caveolae are absent in caveolin-1 knockout mice [36]. While some of the genetic changes in SHR relative to WKY might have arisen through decades of isolation from the parent WKY strain following inbreeding, coordinate changes in gene expression between the SHR and the ACTH-treated WKY rats cannot be attributed to such a cause. Caveolae are now recognized as important sites for colocalisation of signaling molecules, including receptor tyrosine kinases, G-protein-coupled receptors and G-proteins, and hence are critical for vascular function [37]. The inactivation of eNOS through binding to caveolin-1 is the best characterized function for caveolae in endothelial cells [37], highlighting their crucial role in vascular homeostasis. Alterations to caveolae in smooth muscle cells will also impact on cell signalling and recent studies of idiopathic pulmonary arterial hypertension in humans have concluded that increased smooth muscle expression of caveolin-1 contributes to the pathophysiology of the disease through increased capacitative calcium entry [38]. Whether upregulation of caveolin-1 in smooth muscle cells and in endothelial cells could serve to induce hypertension warrants further investigation.

In contrast to caveolin-1, the G protein regulators, Rgs2 and Rgs5, were coordinately downregulated in both SHR and ACTH saphenous arteries. These molecules terminate G protein signalling through Gq and Gi/o by activating the GTPase activity of the Gα subunit. Downregulation thus potentiates the effect of vasoconstrictors like endothelin and angiotensin II [39]. A role in blood pressure regulation has been demonstrated for Rgs2, since knockout mice are severely hypertensive [40]. Activation of Rgs2 occurs in resistance and conduit arteries through binding of protein kinase G, the downstream mediator of nitric oxide action [40,41]. Interestingly, Rgs2 was also decreased in aortic smooth muscle during L-NAME-induced hypertension [42]. Upregulation of caveolin-1 and decreased eNOS function may therefore play a role in the downregulation of Rgs2.

Less is known about a role in blood pressure regulation for Rgs5, which has been described as a marker of arterial but not venous smooth muscle cells [43] and is downregulated during proliferation to form atherosclerotic plaques [44]. In contrast, upregulation of Rgs5 has been reported during neovascularisation of tumours [45]. Rgs5 has also been identified in endothelial cells of cerebral capillaries where it was downregulated in stroke-prone SHR compared to SHR [46]. Our results demonstrating downregulation in arteries from both SHR and ACTH rats would suggest a role in hypertension rather than proliferation since smooth muscle hypertrophy was not a feature of the ACTH model.

## Conclusion

Study of changes in gene expression in two different models of hypertension have highlighted numerous differences which can be linked to variation in the form of vascular remodeling and the unique signalling pathways affecting contractility and cell growth. In contrast, we have identified common changes to upstream targets crucial for the alteration in the balance between vasoconstriction and vasodilation as occurs in hypertension. Common increases in expression of caveolin-1 are likely to increase vasoconstriction through direct effects on calcium influx in smooth muscle cells and indirect inhibitory effects on eNOS in endothelial cells. Decreases in Rgs2 and Rgs5 will augment these effects by prolonging the actions of Gq and Gi/o and their downstream vasoconstrictor pathways. Further studies examining the functional implications of these data and aimed at reversing these gene changes during the different forms of hypertension are needed to determine whether these molecules could be considered as therapeutic drug targets.

## Methods

Sixteen-week-old male WKY and age-matched SHR were used. These rats were originally from stock derived from the National Institutes of Health, Bethesda, MD. A group of 12-week-old male WKY rats was administered ACTH (0.2 mg/kg/day) subcutaneously for 4 weeks. Systolic blood pressure was measured using tail-cuff plethysmography. All experiments were approved by the Australian National University Animal Experimentation Ethics Committee under guidelines of the National Health and Medical Research Council of Australia.

All analyses were made on the first lateral branch of the saphenous artery. This vessel was defined as a resistance vessel due to its small diameter (<250 μm), since feed arteries and the microcirculation contribute to blood pressure regulation in rats [47].

### Electron microscopy

Ultrastructural studies were conducted on a minimum of 3 arteries each from a different rat, as previously described [48,49]. WKY, ACTH and SHR rats were anaesthetized (i.p., 44 and 8 mg/kg, ketamine and xylazine, respectively) and perfused via the left ventricle with 0.1% BSA, 0.1% NaNO_3 _and 10 U/ml heparin and fixed with 1% paraformaldehyde, 3% glutaraldehyde in 0.1 M sodium cacodylate buffer, with 10 mM betaine, 0.2 mM CaCl and 0.15 M sucrose, pH 7.4, using standard procedures [48]. The first lateral branch of the saphenous artery diverging towards the knee was removed and processed for electron microscopy [48,49]. Serial transverse sections (~100 nm thick) totalling ~5 *μ*m of vessel length were cut from each vessel using 3–4 animals/experimental group. Myoendothelial gap junctions connecting the endothelium to the inner layer of smooth muscle were counted and photographed at ×3,500 to ×60,000 on a Phillips 7100 transmission electron microscope. Vessel circumference at the internal elastic lamina, the number of smooth muscle cell layers and endothelial cell areas were determined for each vessel as previously described [48,49]. Statistical significance was tested using one-way ANOVA followed by Student's *t*-test with Bonferroni correction for multiple groups. A p-value < 0.05 was taken as significant. The incidence of caveolae was assessed in montages of the vessel wall using a minimum of 3 arteries, each from a different rat, for each rat group.

### Immunohistochemistry

Rats were anaesthetized as above and perfused with 2% paraformaldehyde in 0.1 M sodium phosphate buffer. The lateral branch of the saphenous artery was removed from both hind legs. Arteries were processed either as whole mounts or were sectioned at 10 μm. Preparations were incubated overnight at room temperature with rabbit antibodies against human caveolin-1 (Santa Cruz; 1:200 for whole mounts, 1:1000 for sections) followed by 1 hour in Cy3 conjugated anti-rabbit IgG (1:100, Jackson Immunoresearch Laboratories). For whole mounts, optical confocal series were taken throughout the thickness of the endothelium and recombined to a single image. For tissue sections, optical confocal series were taken throughout the section thickness and recombined to a single image. All images were collected using the same pinhole and voltage settings so that comparisons could be made of the intensity and extent of staining in arteries from the different rat groups (3 animals/group). Specificity of the staining was tested by preincubation of the antibody with a ten-fold excess of the immunogen.

### Extraction of total RNA

Rats were anesthetized with ether and killed by decapitation. The saphenous artery branch was removed, stripped of extraneous material and stored in RNAlater (Ambion). Three different samples were prepared for each experimental group, i.e. WKY, SHR and ACTH. The choice of three biological replicates was based on the constraints of limited availability of mRNA and the high costs of microarray experiments. This is a common choice in the microarray literature [50]. Each sample, which comprised arteries from 2–3 animals, was snap frozen in liquid nitrogen and processed using the RNeasy Micro Kit (Qiagen), including 20 min DNAse treatment. Concentrations of RNA were measured using a NanoDrop ND-1000 spectrophotometer (NanoDrop Technologies). RNA quality was monitored using RNA 6000 Pico LabChip kit (Agilent 2100 Bioanalyzer). RNA integrity numbers for the 9 samples ranged from 6.4 to 8.1 [51].

### Preparation of cRNA and hybridization on microarrays

Sample preparation followed the Affymetrix GeneChip Eukaryotic Small Sample Target Labeling Assay Version II available at the website [52]. After first round amplification, the yields of unlabeled cRNA ranged from 2.8–7.8 μg. Following the second round amplification, 600 ng of unlabeled cRNA was used for the second round of cDNA synthesis. Double-stranded cDNA was synthesized using oligo(dT) 24-anchored T7 primer, Superscript III (Invitrogen) and 100 ng total RNA for the first strand followed by DNA polymerase I and T4 DNA polymerase for the second strand. The double-stranded cDNA was precipitated with ethanol, air-dried and used as template for the first round of *in vitro *transcription of cRNA with MEGAscript enzyme mix (Ambion) at 37°C for 7 h. The cRNA was purified (RNeasy, Qiagen) and used as template for the second round of double-stranded cDNA synthesis with random primers (Invitrogen). The double-stranded cDNA product of second round synthesis was purified by ethanol precipitation and used as template for the second round of *in vitro *transcription and labeling of cRNA, using the ENZO BioArray HighYield RNA Transcript Labeling kit (Affymetrix) according to the manufacturer's protocol. The yields of labelled cRNA ranged from 58–193.8 μg. All components were mixed, incubated at 37°C for 7 h, and the biotin-labeled cRNA (1 μg for each sample) was purified (RNeasy, Qiagen) and fragmented prior to hybridisation to Affymetrix GeneChip RAE230A arrays. Hybridization, washing and staining followed the Affymetrix GeneChip Expression Analysis protocols.

### Microarray data analysis

Probeset signal values were calculated using the Statistical Algorithm in the Affymetrix MAS5 software. Raw data were normalised by scaling the global mean method to a TGT value of 150. Quality of all arrays was assessed using MAS5 quality control metrics and software procedures available in R/Bioconductor (Bioconductor version 1.8.0 [53]) and at the following website [54]. Based on these metrics, the quality of all arrays was considered to be satisfactory (see Additional file 15).

One-way ANOVA was carried out on the log_2 _transformed MAS5 signal values for each of the 9098 probesets which were present in all replicates of at least one experimental group (Affymetrix MAS5 Statistical Algorithm). Differences between experimental groups were examined using t-tests (post-hoc contrasts).

### GE-biplot and Heat Map

An ordination method, the GE-biplot [55], based on the singular value decomposition of the data matrix of scaled log_2 _signal values, was obtained using an R function available at the website [54]. The GE-biplot is an unsupervised visualisation tool that facilitates the detection of subsets of genes associated with subsets of microarrays. It provides a genome-wide view of the data, showing genes and microarrays simultaneously. Co-regulated (correlated) genes are located close to each other, with genes having reversed response profiles (negatively correlated) being located on the opposite side of the plot. Genes lying further from the centre have higher variance. Genes which lie close to, or lie in the direction of, a group of samples are relatively upregulated in that group compared with other samples, while those lying in the opposite direction are relatively down-regulated in that group. In a GE-biplot, arrays that have approximately the same relative values of gene expression across probesets cluster together. Further details are described elsewhere [55].

A heat map of the MAS 5.0 signal values of the 50 most significant probesets as evaluated by one way ANOVA was constructed [56]. Hierarchical clustering with complete linkage was used to order the arrays in the columns of the heat map and the rank order of the p-values from the ANOVA was used to order the columns.

### Validation of microarray analysis by quantitative PCR

Quantitative PCR was used to determine the copy numbers of mRNA for 13 genes in the WKY, SHR, and ACTH samples (*n *= 3 for each). Details of the genes, primers and product sizes are given in Table 5. Nine of the genes were selected on the basis that their expression was significantly altered in either SHR or ACTH and that they were of biological interest to the processes involved in hypertension. The three vascular connexins and eNOS, although unaltered in the microarrays, were chosen because of their significance to vascular function and potential involvement in hypertension [57]. Validation of the microarray data was considered to be a significant (p < 0.05) change in normalised PCR copy number in the same direction, or lack of a change in normalised PCR copy number which corresponded to that found in the microarray data.

**Table 5:** Real-time PCR primers for validation of microarray data.

**Affymetrix Probeset ID**	**Gene**	**Product size (bp)**	**Primers Forward Reverse**
1387128_at	Adcy3: adenylate cyclase 3	148	agcacctatatggcagcttctgga
			taagcgtgtccttcatggctagtg
1370131_at	Cav1: caveolin 1	150	gcttcaccaccttcactgtgacaa
			ggaagctcttgatgcacggtacaa
1367600_at	Des: desmin	164	gatcaaccttccgatccagacctt
			gcacttcatgttgttgctgtgtgg
1371166_at	NOS3: endothelial nitric oxide	154	tctggcaagaccgattacacgaca
	synthase, eNOS		gtgaggacttgtccaaacactcca
1386881_at	Igfbp3: insulin-like growth factor-	167	tggaaacaccactgagtctgagga
	binding protein 3		agtcaactttgtagcgctggctgt
1373499_at	Gas5: growth arrest-specific 5	152	ccacctgattcaaactccaccatc
			tctgtgatgggacatctggtggaa
1375857_at	Myof: myoferlin	105	cgctgccgaactatttatccatga
			cagacgtatgtgaggcgagagttc
1371776_at	Pik3r1: phosphatidylinositol 3-	146	ggcgaagtcaaacattgcgtcatc
	kinase regulatory subunit polypeptide 1		gtgacattgagggagtcattgtgc
1387074_at	Rgs2: regulator of G-protein	151	ggtgtacagcctgatggagaacaa
	signaling 2		tgggagacagaatggaatgtcctc
1370228_at	Tf: transferrin	140	cgaacggaaagaacactgctgcat
			gaccacaacatggtttggagcttg
1372002_at	Gja1: connexin43 Cx43	306	gagatgcacctgaagcagattgaa
			gatgttcaaagcgagagacaccaa
1368473_at	Gja5: connexin40 Cx40	236	ggaaagaggtgaacgggaagatt
			cacagccatcataaagacaatgaa
1370023_at	Gja4: connexin37 Cx37	131	agctctgcatccaagaagcagta
			agttgtctctcaagtgcctttga
-	18S rRNA GenBank acc. no.	110	ccagtagcatatgcttgtctcaa
	X01117		cgaccaaaggaaccataactgatt

Messenger RNA of all 9 saphenous artery samples was reverse transcribed to cDNA (42°C 1 h, 50°C 1 h, 90°C 10 min) using oligo dT (500 ng.μl^-1^, Invitrogen) primers and Superscript II (200 U.μl^-1^, Invitrogen). For every sample, control reactions from which either reverse transcriptase or RNA was omitted were run in parallel. Each 25 μl PCR reaction mixture contained 1 μl of each primer (final concentration 800 nM), 2 μl of 12.5 mM dNTPs, 3 μl of 25 mM MgCl_2_, 0.125 μl of AmpliTaq Gold (5 U. μl^-1^), 2.5 μl of 10× SYBR Green buffer (SYBR Green Core Reagents kit, Applied Biosystems), 10.375 μl H_2_O and 5 μl of template containing either 10 ng of cDNA or diluted plasmid DNA containing the relevant cloned PCR product. All samples were diluted in water containing tRNA (2 ng. μl^-1^). All reactions were performed in duplicate (ABI Prism 7700 Sequence Detection System, ACRF Biomolecular Resource Facility) at 95°C for 10 min, and 40 cycles of 20 sec at 95°C, 20 sec at 65°C, 45 sec at 72°C. Every experiment included duplicate control reactions and the expression of all genes was determined in every sample. The integrity of each PCR reaction was checked using dissociation curve analysis after every reaction.

For each gene, the mRNA concentration was determined by comparing the fluorescent signal at threshold (equal to 10 × S.D. of baseline fluorescence) to that generated by a standard curve using serial dilutions of a known concentration of purified plasmid DNA containing the relevant cloned and sequenced PCR product. Sequences were obtained using an ABI 3730 sequencer (ACRF Biomolecular Resource Facility, JCSMR, ANU). The concentration of each mRNA was normalized by determining 18S rRNA concentration, using primers complementary to a region of the 5' end of the molecule and a standard curve using serial dilutions of plasmid DNA containing the cloned fragment of the 18S rRNA gene [29]. Results were analyzed for statistical significance (p < 0.05) using ANOVA and Student's t-test.

### Direct interactions between selected genes

To examine differences in the major pathways affected in SHR and ACTH-induced hypertension, genes that were identified by t-tests as significantly altered in expression in SHR or ACTH compared with WKY (at the 0.015 level) and that were significant in ANOVA at the 0.02 level, were imported into a demonstration version of PathwayStudio 4.0 (Ariadne Genomics, Rockville, MD). This software contains the proprietary ResNet 3.0 database of functional relationships between genes, proteins and small molecules automatically extracted from PubMed and scientific journals using the MedScan natural language processing software. Functional links between genes and proteins were also investigated and cross checked using publicly available gene and genome databases: Online Mendelian Inheritance in Man (OMIM) and Genomic Biology and software provided by NCBI [58], and the protein database Human Protein Reference Database (HPRD; [59]) and software [60].

## Authors' contributions

THG extracted RNA, performed quantitative PCR, contributed to the analysis and interpretation of microarray data, analysed biological association networks and interactions between genes and proteins, and drafted the manuscript. SJO performed quality assessment of the microarrays, contributed to the design and statistical analyses of the microarray data, prepared data for the figures, participated in interpretation of the results and contributed to writing the manuscript. TDB administered ACTH, measured blood pressure of experimental rat groups and performed serial section electron microscopy and morphometric measurements. KRM performed the immunohistochemistry and measured blood pressure of experimental animals. KP amplified and labeled mRNA and conducted all of the microarray hybridizations. YEP and SRW designed and performed statistical analyses of data and contributed to the interpretation of results and preparation of the manuscript. SLS performed serial section electron microscopy and morphometric measurements, assisted with experimental design and contributed to preparation of the manuscript. CEH conceived of the study and coordinated the experimental design, dissected arteries, contributed to the interpretation of data and made a substantial contribution to the writing of the manuscript. All authors have read and approved submission of the manuscript.

## Supplementary Material

Additional file 1 Genes with increased expression in ACTH vs WKY and unchanged in SHR vs WKY. Gene lists of statistical analysis of microarray data including gene names, gene descriptions, biological function, Affymetrix IDs, accession numbers, expression levels and p-values. Click here for file

Additional file 2 Genes with decreased expression in ACTH vs WKY and unchanged in SHR vs WKY. Gene lists of statistical analysis of microarray data including gene names, gene descriptions, biological function, Affymetrix IDs, accession numbers, expression levels and p-values. Click here for file

Additional file 3 Genes with increased expression in SHR vs WKY and unchanged in ACTH vs WKY. Gene lists of statistical analysis of microarray data including gene names, gene descriptions, biological function, Affymetrix IDs, accession numbers, expression levels and p-values. Click here for file

Additional file 4 Genes with decreased expression in SHR vs WKY and unchanged in ACTH vs WKY. Description of dataset: Gene lists of statistical analysis of microarray data including gene names, gene descriptions, biological function, Affymetrix IDs, accession numbers, expression levels and p-values. Click here for file

Additional file 5 Genes with increased expression in ACTH and SHR vs WKY. Gene lists of statistical analysis of microarray data including gene names, gene descriptions, biological function, Affymetrix IDs, accession numbers, expression levels and p-values. Click here for file

Additional file 6 Genes with decreased expression in ACTH and SHR vs WKY. Gene lists of statistical analysis of microarray data including gene names, gene descriptions, biological function, Affymetrix IDs, accession numbers, expression levels and p-values. Click here for file

Additional file 7 Genes with increased expression in ACTH vs WKY and decreased expression in SHR vs WKY. Gene lists of statistical analysis of microarray data including gene names, gene descriptions, biological function, Affymetrix IDs, accession numbers, expression levels and p-values. Click here for file

Additional file 8 Genes with decreased expression in ACTH vs WKY and increased expression in SHR vs WKY. Gene lists of statistical analysis of microarray data including gene names, gene descriptions, biological function, Affymetrix IDs, accession numbers, expression levels and p-values. Click here for file

Additional file 9 Genes with increased expression in ACTH vs WKY. Gene lists of statistical analysis of microarray data including gene names, gene descriptions, biological function, Affymetrix IDs, accession numbers, expression levels and p-values. Click here for file

Additional file 10 Genes with decreased expression in ACTH vs WKY. Gene lists of statistical analysis of microarray data including gene names, gene descriptions, biological function, Affymetrix IDs, accession numbers, expression levels and p-values. Click here for file

Additional file 11 Genes with increased expression in SHR vs WKY. Gene lists of statistical analysis of microarray data including gene names, gene descriptions, biological function, Affymetrix IDs, accession numbers, expression levels and p-values. Click here for file

Additional file 12 Genes with decreased expression in SHR vs WKY. Gene lists of statistical analysis of microarray data including gene names, gene descriptions, biological function, Affymetrix IDs, accession numbers, expression levels and p-values. Click here for file

Additional file 13 Genes with increased expression in SHR vs ACTH. Gene lists of statistical analysis of microarray data including gene names, gene descriptions, biological function, Affymetrix IDs, accession numbers, expression levels and p-values. Click here for file

Additional file 14 Genes with decreased expression in SHR vs ACTH. Gene lists of statistical analysis of microarray data including gene names, gene descriptions, biological function, Affymetrix IDs, accession numbers, expression levels and p-values. Click here for file

Additional file 15 Density plots and boxplots of log_2 _CEL file intensities. A. The density plots showed the expected right-skewed distribution. There was no evidence of scanner saturation. B. Boxplots showed a consistent shape and size which indicated consistency between samples. Click here for file
