# Supplementary figures and images for: Vascular microarray profiling in two models of hypertension identifies caveolin-1, Rgs2 and Rgs5 as antihypertensive targets
Source: BMC Genomics. 2007 Nov 7;8:404. doi: 10.1186/1471-2164-8-404 (PMC2219888; doi:10.1186/1471-2164-8-404)

**A**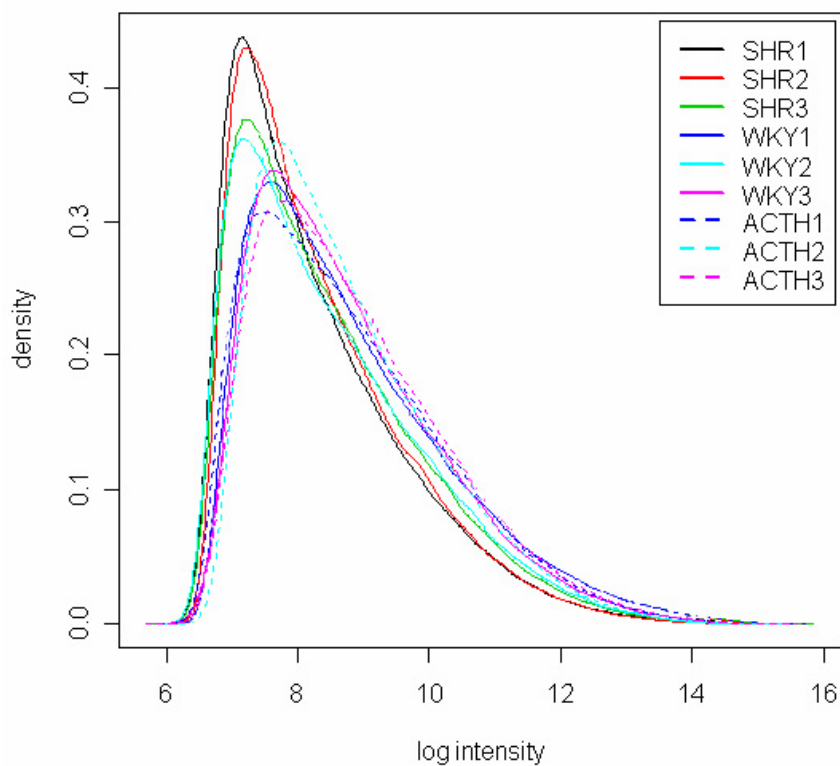**B**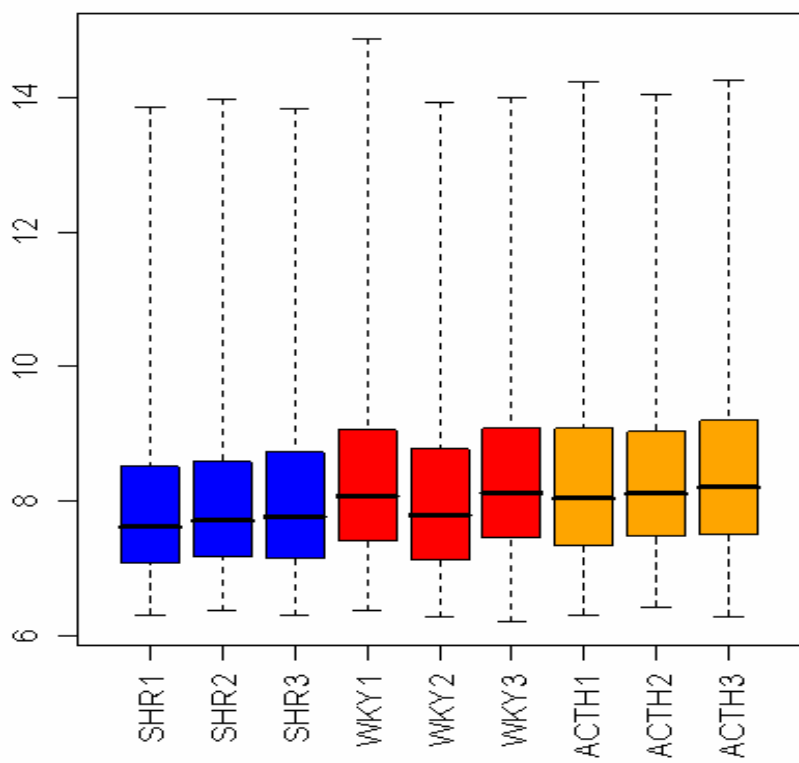

Supplement: Additional file 15 — Density plots and boxplots of log2 CEL file intensities. A. The density plots showed the expected right-skewed distribution. There was no evidence of scanner saturation. B. Boxplots showed a consistent shape and size which indicated consistency between samples. [file 1471-2164-8-404-S15.pdf]
